# Case Report: Complete response to pembrolizumab monotherapy in a geriatric patient with metastatic Merkel cell carcinoma and TNBC

**DOI:** 10.3389/fonc.2025.1579287

**Published:** 2025-08-27

**Authors:** Jessica Liu, Kelly Olino, Maryam Lustberg, Jane Kanowitz

**Affiliations:** ^1^ Yale University, New Haven, CT, United States; ^2^ Yale Cancer Center, Yale School of Medicine, New Haven, CT, United States; ^3^ Smilow Cancer Hospital, New Haven, CT, United States

**Keywords:** immune-checkpoint inhibitors, merkel cell carcinoma, triple-negative breast cancer, geriatric oncology, immune-related adverse events, pembrolizumab

## Abstract

We present a case of a 98-year-old patient with metastatic Merkel cell carcinoma (MCC), a rare and aggressive neuroendocrine skin cancer, and locally advanced triple-negative breast cancer (TNBC), who achieved durable remission of both diseases with anti-PD-1 pembrolizumab monotherapy. This case is particularly significant to clinical management of advanced cutaneous malignancy in the elderly, who are historically underrepresented in clinical trials, as it demonstrates the remarkable efficacy of pembrolizumab alone while avoiding toxicities associated with traditional chemotherapy. The regression of the MCC lesion is noteworthy given the typically poor prognosis of metastatic MCC in geriatric patients. This experience contributes to the growing body of evidence supporting immunotherapy as a well-tolerated option for older adult patients with advanced skin and/or breast cancers, particularly when careful toxicity screening is employed for patient selection.

## Introduction

The anti-PD-1 immune checkpoint inhibitor (ICI) pembrolizumab blocks the T-cell-inhibitory programmed cell death protein 1 (PD-1) pathway and has gained tumor agnostic approval for treatment of high mutational burden tumors and tumors expressing programmed death ligand 1 (PD-L1) (1, 2). Triple negative metastatic breast cancer (TNBC) is a highly aggressive subtype of breast cancer; In combination with chemotherapy, pembrolizumab has improved overall survival in patients with metastatic TNBC (3). Similarly, pembrolizumab has demonstrated tumor control against the neuroendocrine Merkel cell carcinoma (MCC), a rare and aggressive skin cancer (4–6). With an incidence of 0.66 cases per 100,000 in the U.S. and peak occurrence in patients aged 75–85 years, MCC primarily affects elderly populations (7). Despite the advanced age of most patients with MCC, data on pembrolizumab monotherapy efficacy and immune-related adverse event (irAE) tolerability remain limited in patients over 75 years or those with poor Eastern Cooperative Oncology Group (ECOG) performance status (PS > 1). While the KEYNOTE-017 trial of pembrolizumab in MCC patients did not find a significant difference in efficacy between patients younger versus older than 70 years, others have suggested that the elderly population’s higher incidence of cancer, reduced T-cell reserves, and poor tolerance of iRAEs may lead to worse real-world outcomes of ICIs (5, 8, 9).

However, we present a case of a 98-year-old patient with metastatic MCC and locally advanced TNBC who achieved durable remission of both diseases on anti-PD-1 ICI pembrolizumab alone. This case provides real-clinic evidence for pembrolizumab monotherapy tolerability in patients older than 75 years old and with multiple, advanced malignances.

## Case description

**Figure 1:**
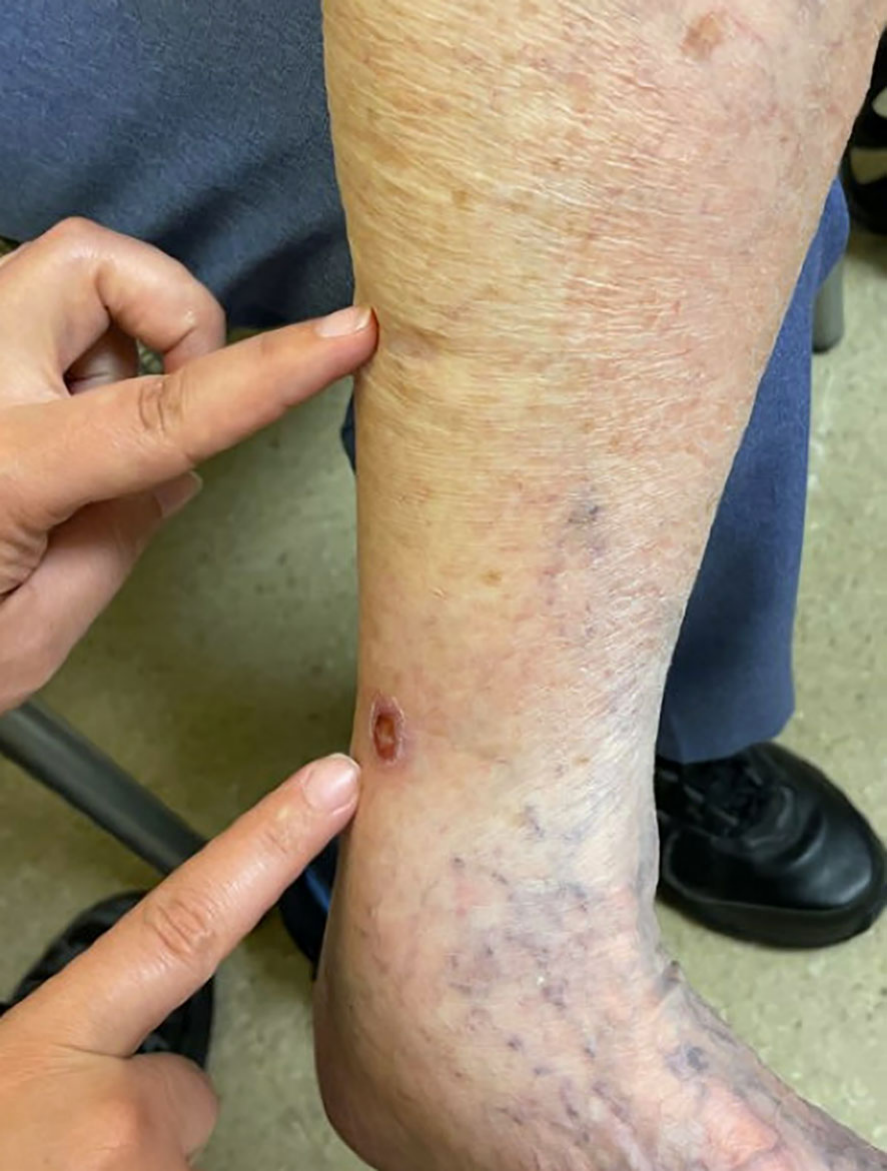
Skin lesions on the left ankle of the patient. The lesion treated with radiotherapy in 2022 was the more superior lesion (indicated by the top pointer finger), while the lesion also diagnosed as MCC in 2023 was the more distal lesion (indicated by the lower pointer finger).

A 98-year-old woman presented with a skin mass in the lower extremity in May 2022. The patient had multiple medical comorbidities with hypertension, chronic renal insufficiency, hypercholesterolemia, aortic stenosis, bradycardia with syncope, for which she had a cardiac pacemaker placed just prior to diagnosis. Immunohistochemistry of the skin lesion reported the lesion as CK20 negative, TTF-1 negative, synaptophysin positive, and INSM1 positive, supporting the diagnosis of Merkel cell carcinoma. Given her overall frailty and ECOG PS of 2 at the time of diagnosis, small tumor size (1cm), and location on the medial aspect of the left ankle (**Figure 1**), the patient opted for one dose of 8Gy radiation in August 2022 over systemic chemotherapy or immunotherapy, prioritizing a localized treatment approach for the limited disease burden at initial presentation. Radiotherapy led to complete regression of the lesion.

## Diagnostic assessment

**Figure 2:**
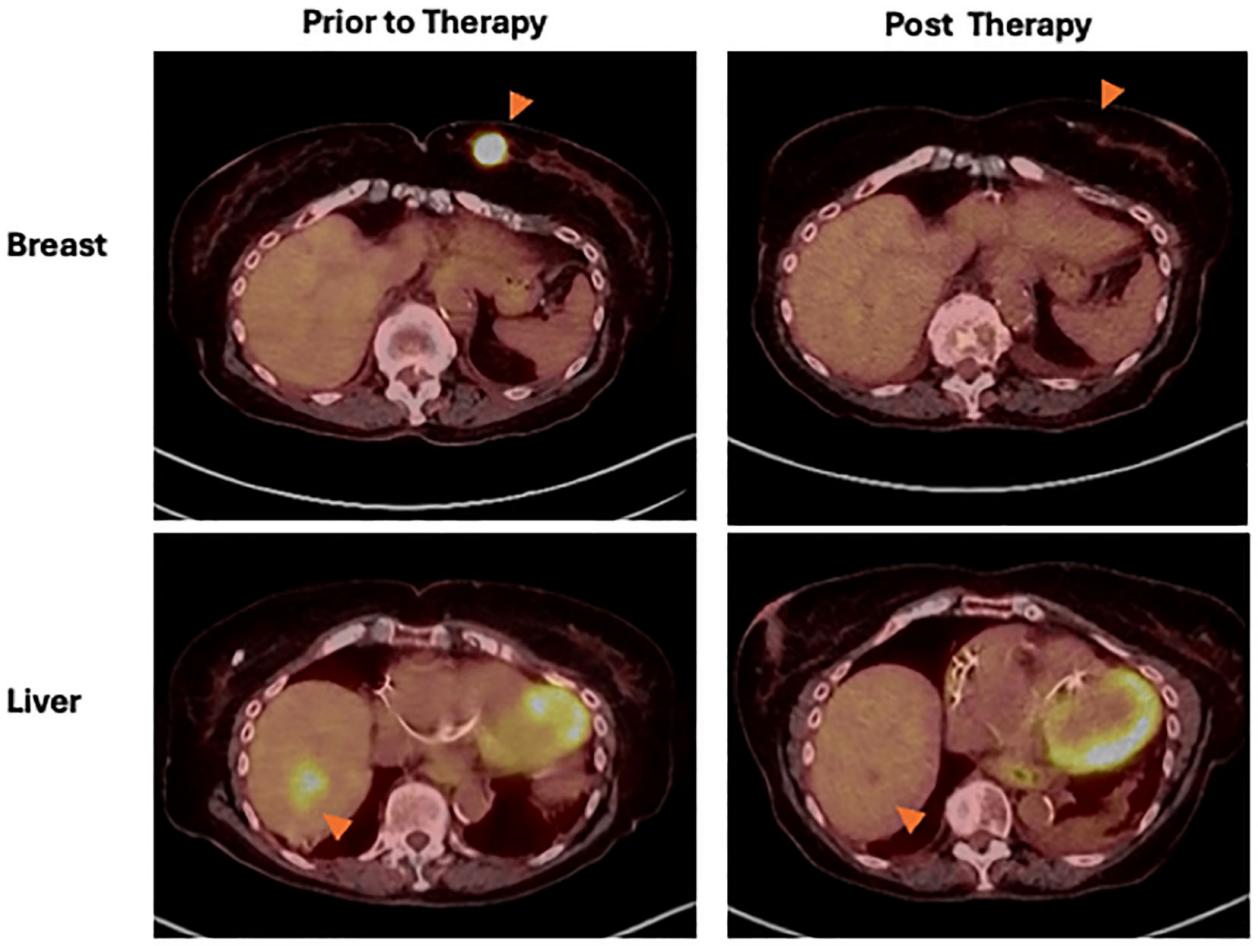
Breast mass (top row) and liver lesion (bottom row) indicated by orange arrowheads. PET CT demonstrates metabolic response of both lesions following pembrolizumab treatment in March 2024 compared to before treatment in September 2023.

In September 2023, her disease recurred, and biopsy showed in-transit Merkel cell carcinoma. Her ECOG PS had improved to one, and the patient underwent PET CT for staging. Imaging revealed a 3cm lesion in the right lobe of the liver and a 3cm hypermetabolic left breast mass (**Figure 2**). Biopsy of the liver mass revealed metastatic MCC. Breast biopsy identified a poorly differentiated triple negative invasive ductal carcinoma mass, stage IIB with PD-L1 combined positive score (CPS) > 10 by pathology. To assess the patient’s risk of grade 3 or higher toxicity to chemotherapy, the validated CARG (Cancer and Aging Research Group) chemotherapy toxicity calculator was utilized (10), with her score of 15 indicating a 92% risk of grade 3 or higher toxicity.

Given the synchronous diagnosis of two cancers and concern for chemotherapy related toxicity, pembrolizumab (anti-PD-1 ICI) was recommended. Pembrolizumab was selected over other ICIs based on the breast tumor’s PD-L1 CPS > 10, which suggested favorable response to immunotherapy, and the more extensive literature supported pembrolizumab use across multiple tumor types (3, 5, 11).

## Therapeutic intervention

Beginning in November 2023 and until February 2024, the patient received four treatments of pembrolizumab 200mg i.v. every 3 weeks without toxicity.

## Outcome

After the first cycle, her breast mass and skin lesions were no longer palpable. PET and clinical examination in March 2024 demonstrated complete imaging and clinical remission of all sites of disease, as shown in **Figure 2**. The patient remained active and ambulatory with a walker. Six months after completion of pembrolizumab monotherapy, however, a new lesion at the L3 vertebra was observed in late August 2024, requiring palliative radiation therapy and kyphoplasty. In mid-December, the patient was hospitalized with congestive heart failure complicated by Influenza B (pneumonia), and she passed in January 2025.

**Figure 3:**
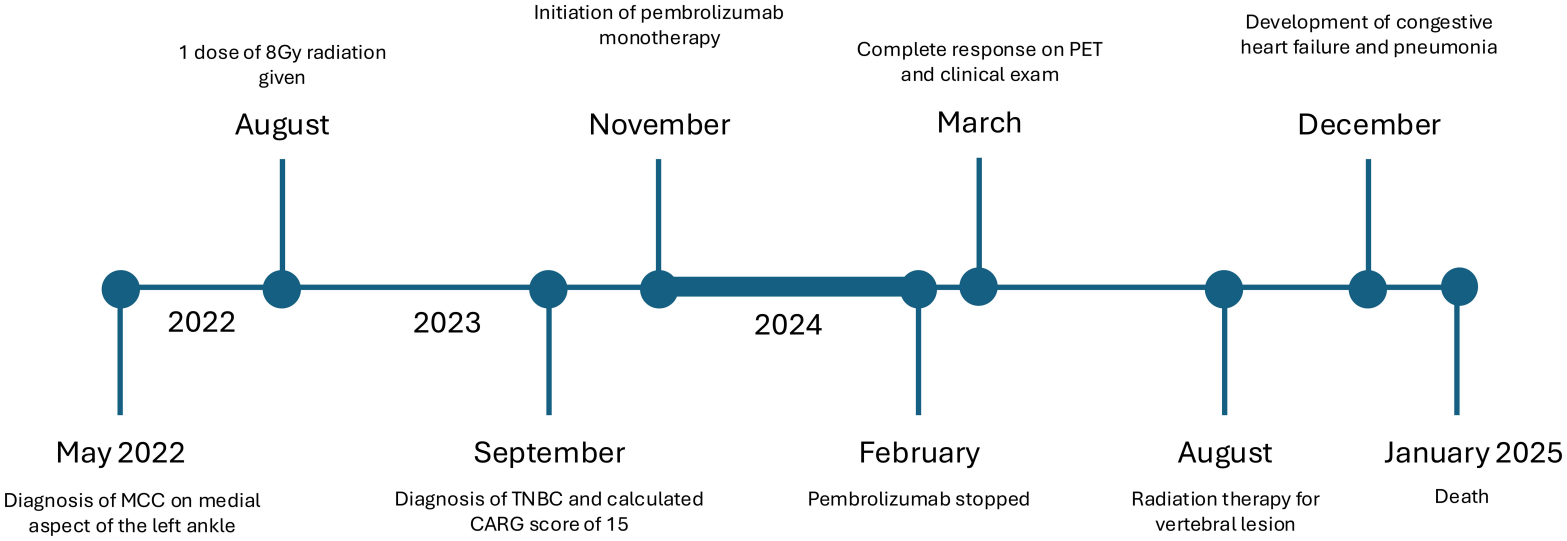
Timeline of the clinical course of the patient. Bolded line indicates the treatment period of pembrolizumab monotherapy.

Nevertheless, this case highlights the successful use of immunotherapy in an elderly patient who presented with concurrent diagnoses of high-grade TNBC and metastatic MCC with visceral involvement, achieving durable remission and maintaining disease control for a significant duration. **Figure 3** presents the patient care timeline.

## Discussion

Both advanced MCC and TNBC are rare and aggressive carcinomas that have been found to respond to ICI antibodies against PD-1 and PD-L1. Cancer cells can evade immune response by expressing PD-L1, which binds T-cell PD-1 receptor and subsequently activates the PD-1 immune checkpoint pathway that blocks T-cell activation (2). Both PD-1 and PD-L1 ICIs have been shown to block PD-1 pathway signaling, promoting antitumor immune response in patients with metastatic MCC (4). Yet, over-activation of the immune response can lead to irAEs such as dermatitis and colitis/diarrhea, with over 30% of pembrolizumab clinical trial patients with MCC experiencing at least one grade 3 or higher irAE (11, 12). While recent multicenter, prospective studies did not find a correlation between age and irAE occurrence, patients of ages greater than 75 years exhibit less tolerance of and are more likely to discontinue treatment due to irAEs (9, 12, 13). The underrepresentation of patients older than 75 years and/or with PS > 1 in clinical trials also suggests that tolerability of ICIs in the geriatric patient population may be over-estimated. Therefore, assessment of functional status, comorbidities, and psychosocial factors is increasingly important when selecting geriatric patients for immunotherapy.

Despite the rarity of this dual malignancy presentation and the patient’s advanced age of 98 years, this case demonstrates broadly applicable principles for geriatric oncology patients. Considering the increasing incidence of MCC in patients older than 70 (7), the use of the CARG toxicity risk score (8) in this case provides a validated framework for treatment decision-making with geriatric oncology patients. More broadly, for patients with single advanced malignancies expressing PD-L1 representing approximately 20% of TNBC (14) and 50% of Merkel-cell carcinomas (15), this risk-stratification methodology can guide clinicians in weighing chemotherapy toxicity against immunotherapy benefits. In our case, the patient’s PS > 1 and CARG score of 15 made chemotherapy an unfavorable palliative option. Rather, the patient’s high risk of chemotherapy toxicity and the breast tumor’s PD-L1 CPS > 10 supported the decision to initiate pembrolizumab treatment.

The patient’s onset of heart failure and pneumonia raises consideration of whether pembrolizumab may have contributed indirectly through subclinical immune effects, however, our patient exhibited no acute irAEs and maintained her ECOG performance status of 1 throughout treatment.

Even with our patient’s advanced age, her response compares favorably to outcomes reported in the KEYNOTE-017 trial, which demonstrated a 58% overall response rate and 30% complete response rate in advanced MCC patients with a median age of 70.5 years (5). Her durable response with pembrolizumab alone presents a remarkable case of PD-1 ICI control of rare metastatic MCC and advanced TNBC, suggesting that advanced age alone may not preclude favorable outcomes with pembrolizumab in carefully selected patients.

Therefore, this case provides real-world evidence that with geriatric patient-specific evaluation of the risk of chemotherapy toxicity, PD-1 inhibitor immunotherapy can be effective, well tolerated, and may be considered in adults older than 75 years having PD-L1-expressing tumors.

## Data Availability

The original contributions presented in the study are included in the article/Supplementary Material. Further inquiries can be directed to the corresponding author.
